# Organoids from the Human Fetal and Adult Pancreas

**DOI:** 10.1007/s11892-019-1261-z

**Published:** 2019-12-11

**Authors:** Jeetindra R. A. Balak, Juri Juksar, Françoise Carlotti, Antonio Lo Nigro, Eelco J. P. de Koning

**Affiliations:** 1 Department of Internal Medicine, Nephrology, Leiden University Medical Center, Albinusdreef 2, 2333 ZA Leiden, The Netherlands; 2 Hubrecht Institute for Developmental Biology and Stem Cell Research, Utrecht, The Netherlands

**Keywords:** Organoids, 3D culture, Pancreas, Stem cells, Regeneration, Development, Diabetes Mellitus, Type 1

## Abstract

**Purpose of Review:**

Novel 3D organoid culture techniques have enabled long-term expansion of pancreatic tissue. This review comprehensively summarizes and evaluates the applications of primary tissue–derived pancreatic organoids in regenerative studies, disease modelling, and personalized medicine.

**Recent Findings:**

Organoids derived from human fetal and adult pancreatic tissue have been used to study pancreas development and repair. Generated adult human pancreatic organoids harbor the capacity for clonal expansion and endocrine cell formation. In addition, organoids have been generated from human pancreatic ductal adenocarcinoma in order to study tumor behavior and assess drug responses.

**Summary:**

Pancreatic organoids constitute an important translational bridge between in vitro and in vivo models, enhancing our understanding of pancreatic cell biology. Current applications for pancreatic organoid technology include studies on tissue regeneration, disease modelling, and drug screening.

## Introduction

The pancreas is a mixed endocrine and exocrine gland that plays a pivotal role in digestion and metabolic homeostasis. The endocrine pancreatic compartment includes the alpha and beta cells which secrete the hormones glucagon and insulin, respectively, that are required for normal glycemic control. The exocrine compartment is composed of acinar cells, which store and secrete digestive enzymes that are drained into the duodenum by a highly branched, tubular epithelial tree–like duct network within the pancreas.

Pancreas biology has been intensively studied, in an effort to understand pancreas development and function, to obtain insight into pathophysiological processes, to identify disease-associated markers, and to find better treatment options for devastating pancreatic diseases associated with high mortality and health care costs, such as diabetes mellitus, pancreatic adenocarcinoma (PDAC), pancreatitis, and cystic fibrosis. Studying the normal human pancreas at the cellular level is difficult. The high risk for complications of pancreatic biopsies more or less precludes this option to generate cross-sectional and longitudinal data of normal human pancreatic tissue at the cellular level in most patients. So we are dependent on generating cross-sectional data from surgical specimens, being aware that the pathological process for which the surgery was indicated could affect the “normal” pancreatic tissue surrounding the pathological process, or from donor pancreas that is not (entirely) used for pancreas or islet transplantation. In vivo models, such as rodents, do not have this limitation but are time consuming and expensive. Moreover, results obtained from in vivo animal models are not always translatable to humans [1]. Although characterized by simplicity and good controllability, two-dimensional (2D) culture models using immortalized cell lines or primary cells limit the study of crucial aspects such as cell polarity, cell to cell contacts, three-dimensional self-organization, and interaction with stromal cells and other extracellular matrix components [2–6]. Moreover, adaptation of primary cells or cell lines during monolayer culture conditions fundamentally changes cell behavior so that findings are often no longer translatable to the primary tissue [7–9].

Recent breakthroughs in three-dimensional (3D) culture methods have led to the development of organoid culture platforms, which allow researchers to perform translational research on long-term in vitro cultures not limited by barriers present in 2D culture or animal models [10–13]. Organoids are three-dimensional cellular structures created through self-organization of cells that can closely mimic the architecture and functionality of the native organ from which the cells were originally derived.

Long-term organoid cultures were originally established by the identification of conditions that recapitulate the in vivo intestinal stem cell niche in vitro. The field was spearheaded by a report that organoids could be made from isolated mouse intestinal crypts cultured in Matrigel and supplemented with growth factors that stimulated Wnt signalling, reflecting the high degree of Wnt signalling in intestinal crypts [14]. One of the key components in organoid culture systems is the 3D environment in which cells behave most optimally. Cells are cultured within an extracellular matrix, such as Matrigel, which prevents attachment of cells to the surface of the tissue culture plates and aims to mimic in vivo mechanical and biochemical stimuli dictating cell polarization and autonomous reorganization [15–18]. In addition to the extracellular matrix, organoids are cultured in a medium that usually contains growth factors that are known to stimulate proliferation during organogenesis and tissue homeostasis, such as Wnt activators (Wnt3a, R-spondin), receptor tyrosine kinase ligands (EGF, FGF10), BMP inhibitors (Noggin), and TGF-beta inhibitors [19]. The starting material of organoids can either be fragmented or dissociated primary tissue, or pluripotent cells such as embryonic stem cells (ESC) or induced pluripotent stem cells (iPSC) that are pre-differentiated in vitro. One of the additional advantages of organoids is the large amount of biomass that can be expanded with just one or few progenitor cells.

In this review, we discuss the application of human fetal and adult tissue–derived pancreatic organoids in regenerative studies and their use as patient-specific tools for disease modelling.

### Generation of Organoids from Fetal Pancreatic Tissue: Fetal Pancreatic Organoids

Pancreas organogenesis is a complex process requiring a dynamic spatiotemporal interplay of multiple cells and their surrounding niche. Deciphering the molecular mechanisms underlying morphogenesis and cell specification is essential for a better understanding of pancreas development and regeneration. While the use of transgenic animal models has greatly increased our knowledge of the molecular basis of pancreatic lineage decisions and cell specification, the mechanisms governing morphogenesis are not well understood. Thus, a different approach for the study of fetal development is necessary. Because there are currently no cell lines available that can act as multipotent pancreatic progenitor cells, an alternative approach to overcome this hurdle is the culture of primary fetal tissue.

Due to the limited availability of human fetal tissue, rodent fetal tissue has initially been used for in vitro studies. Some of the early insights into rodent pancreatic differentiation arose from the culture of rat fetal pancreas explants, with the tissue being cultured with or without the surrounding mesenchyme. A marked increase in endocrine cells was observed in cultures without mesenchymal cells [20, 21].

While rodent explants at least partially recapitulate endocrine differentiation in vitro, they are limited by their short-term maintenance and the contribution of multiple cell types, which makes it difficult to characterize and study the bona fide fetal pancreatic stem cells. Prior studies indicated that cells expressing SOX9 during early pancreas development can give rise to both endocrine and exocrine cells making this a suitable candidate marker for multipotent progenitors [22]. With that in mind, SOX9 progenitor cells that were negative for the endocrine progenitor marker Ngn3 were isolated by flow cytometry from E11.5 SOX9-eGFP, NGN3-tdTomato mouse pancreas and embedded in Matrigel for clonal expansion [23]. These single cells generated heterogeneous spheres with SOX9(+), NGN3(−) cells, and a small amount of SOX9(−), NGN3(+), C-peptide(+), and glucagon(+) cells in vitro, showing a degree of multipotency which was maintained for up to three passages. Additional shRNA-based loss-of-function screening of endocrine developmental genes was performed on these spheres to demonstrate that these spheres recapitulate several key aspects of endocrine differentiation in vitro, thus establishing a possible model for the study of differentiation of multipotent progenitor cells in vitro*.*

**Fig. 1:**
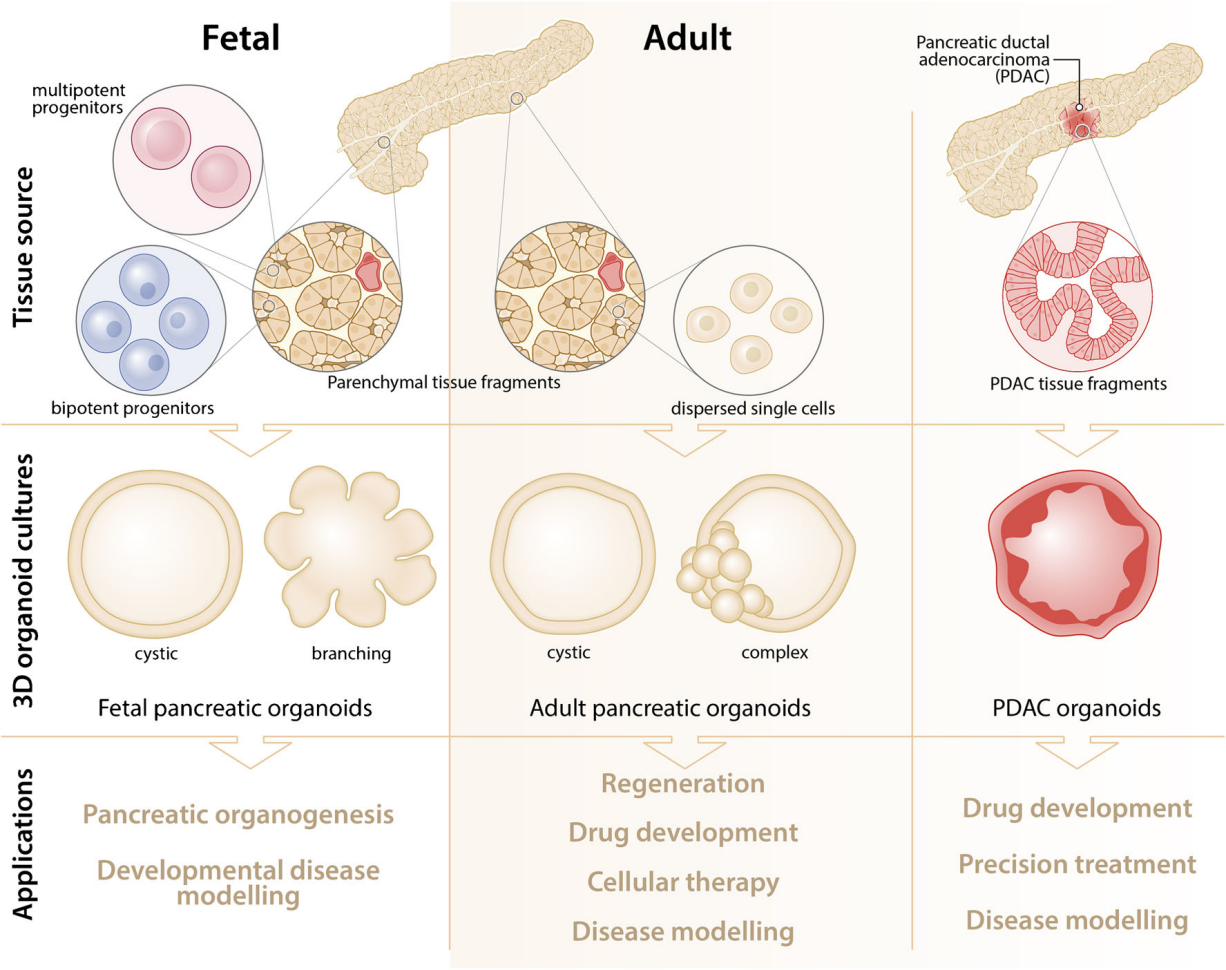
Pancreatic organoid generation and its applications. By using 3D culture conditions, it is possible to generate organoids from primary fetal and adult pancreatic tissue or from pancreatic ductal adenocarcinoma (PDAC). Depending on the starting tissue/cell source and the culture conditions, there are differences in organoid morphology and cellular composition. More branching fetal organoids can be generated from fragments of fetal pancreatic parenchymal tissue. Dispersed single cells from these fetal tissue fragments can give rise to cystic organoids with multipotent progenitors also generating branching organoids. They permit the study of developmental cues and morphogenesis. Similarly, adult pancreatic parenchymal tissue fragments can lead to the formation of more complex organoids while dispersed single cells generate more cystic organoids, allowing the study of regenerative mechanisms, drug development, modelling of disease, or differentiation towards endocrine lineages. Tissue biopsies from patients with pancreatic ductal adenocarcinoma (PDAC) can be used to produce patient-specific PDAC organoid lines that can be used for disease modelling, drug development, and person-centered treatment options (precision medicine)

A similar method was applied in an effort to investigate the morphogenesis of murine pancreatic duct development [24]. Cells expressing SOX9 were isolated from E10.5 SOX9-eGFP mice and cultured in Matrigel. Depending on the specific combinations of growth factors used in the culture medium two different types of organoids could be generated—hollow spheres which maintained a higher contribution of progenitor cells that were similar to the previously described cells [23], or complex organoids containing more differentiated cells (Fig. 1). These complex organoids developed a branching ductal network of polarized cells and showed tip-trunk segregation reminiscent of pancreas morphogenesis in vivo. These organoids were shown to grow out from their tip-like structures forming tubular networks, the branching of which is thought to be caused by strong local inhibitory signalling [25, 26]. As such, the tips of these organoids formed acinar cells, whereas the center of the organoids contained HNF1β(+), SOX9(+), and PDX1(+) progenitor–like cells and a small number of endocrine cells. Furthermore, endocrine cell formation in these organoids could be increased through inhibition of mesenchymal signalling by the removal of FGF1 from the culture medium or by blocking FGF signalling via small molecule inhibitors [24]. To study the interplay between mesenchymal signalling and organoid differentiation, a co-culture method was developed in which E10.5 mouse organoids were tightly enveloped by their native mesenchyme [27]. Using these organoids, it was shown that the gene NFIA acts as a regulator of Notch activation through blockade of Dll1 endocytosis leading to an endocrine fate [28]. Interestingly, these results obtained with organoids are in line with the results from rat fetal explant cultures, in which the presence of mesenchyme was associated with reduced endocrine cell specification.

In an effort to obtain insight into human fetal pancreas development, Bonfanti et al. [29•] used human fetal pancreatic tissue to create a model for the study of pancreas development in a 3D culture system. The authors described efficient expansion of small fragments of human fetal tissue that, similar to their mouse counterparts, grew as hollow spheres containing polarized cells. These fetal organoids also expressed the key transcription factors of pancreatic progenitor cells (PDX1, NKX6.1, SOX9). By using a growth factor combination of the Wnt agonist R-Spondin1 (RSPO1), FGF10 and EGF, they were able to keep these progenitors in expansion for up to 5 months. Interestingly, in the absence of EGF in the culture medium, endocrine differentiation was promoted at the expense of proliferation.

Although large steps have been taken in the study of pancreatic development through the use of fetal organoids, limited accessibility and ethical considerations hamper the use of human fetal tissue. Nevertheless, the use of human fetal tissue in organoid culture is a valuable method to study signalling pathways and conditions necessary for normal pancreas morphogenesis and cell specification.

### Generation of Organoids from Adult Pancreatic Tissue: Adult Pancreatic Organoids

Recent technological achievements have revealed that adult tissues may contain a more abundant source of tissue stem cells than previously anticipated, allowing organoid cultures from multiple types of tissues to study differentiation, tissue stem cell maintenance and disease. A breakthrough in adult primary tissue culture was the discovery of Lgr5(+) adult stem cells in the intestinal crypts of mice that generated organoids in vitro [14, 30]. Since then, organoid culture methods have been established for stomach, liver, pancreas, brain, lungs, and many other organs and tissues [31]. This led to the hypothesis that the culture of putative tissue stem cells from the adult human pancreas could prove useful for studies into pancreas regeneration, including regeneration of pancreatic islets which could be potentially relevant for beta cell replacement therapy.

Human pancreatic ducts have been shown to exhibit a capacity for in vitro expansion and formation of 3D hollow structures when cultured in collagen or Matrigel, hence showing potential as tools for regenerative studies [32]. Initially the short-term expansion and differentiation capacity of adult human and mouse pancreatic cells was demonstrated in suspension culture, where single islet and duct cells formed spheres expressing both neural and progenitor markers. Single spheres from both origins showed capacity for differentiation towards C-peptide-positive beta-like cells and neurons, demonstrating that a clonally expandable progenitor pool could be present in the adult human and mouse pancreas [33, 34]. To zoom in on the putative progenitor cell in the murine pancreas, Jin et al. used flow cytometry to isolate and culture a subpopulation of ductal cells that formed hollow ductal spheres in a methylcellulose and Matrigel-containing semi-solid medium. These cells showed capacity for differentiation towards an endocrine cell fate after being treated with R-Spondin1, which induced the formation of dense organoids with increased expression of endocrine progenitor markers [35]. Huch et al. [36] used similar culture conditions but applied them on small ductal fragments isolated from mice. These organoids formed hollow spheres of ductal cells that generated budding structures and demonstrated unlimited expansion while maintaining genetic stability. Although these structures retained ductal characteristics in vitro, they were able to form endocrine-like cells when co-transplanted with mouse embryonic pancreatic cells under the kidney capsule of mice, demonstrating that these organoids harbor bipotent progenitors.

Multiple groups have reported on the generation of pancreatic organoids derived from the adult human pancreas [37, 38••]. These organoids formed mostly hollow spheres with no capacity for the formation of complex tip-trunk structures or any spontaneous endocrine cell differentiation, which was observed in fetal tissue–derived organoids (Fig. 1). Human adult tissue–derived organoids could however still be coerced into differentiation towards a beta cell-like phenotype by the overexpression of key transcription factors regulating beta cell formation [37]. Recently, we described a protocol for the generation of more complex organoids derived from human pancreatic islet–depleted tissue fragments [39••] (Fig. 1). Without genetic modification, these organoids showed increased expression of aldehyde dehydrogenase (ALDH) in their budding structures and showed formation of de novo insulin(+) cells upon xenotransplantation into immunodeficient mice. Additionally, we showed that increased ALDH activity in human pancreatic organoids could be used to enrich for cells with colony forming capacity.

Organoids derived from the adult human pancreas differ from fetal pancreatic organoids in multiple aspects, such as morphology and endocrine cell formation, most of which are likely to be explained by the apparent absence of a multipotent progenitor in the adult organ or the lack of an optimal culture medium or enrichment strategies. They are however important tools to study regeneration by identification of the cell population responsible for organoid formation and of factors or cell to cell interactions involved in this process.

### Which Pancreatic Cell Types Generate the Pancreatic Organoids?

Unlike the small intestine, there is limited information on the presence or identity of progenitor cells in the adult pancreas [30] and whether in their absence regeneration and homeostasis is orchestrated through the replication and transdifferentiation of mature cells. The matter is further complicated by contradictory results from animal models. We know that animal models do not fully recapitulate human physiology which is known for its low cell turnover and limited regenerative capacity [40–46], whereas animal models demonstrate increased pancreatic regeneration, beta cell proliferation, and islet neogenesis [22, 47–52]. Nevertheless, human beta cell plasticity has been observed when there is increased insulin demand such as in pregnancy or obesity [53, 54]. Apparently this regenerative capacity fails in patients with type 2 diabetes that have a decreased beta cell mass, despite their increased insulin demand [54–56]. Additionally, patients suffering from pancreatitis, an inflammation of the pancreas, show signs of exocrine metaplasia and ductal cell proliferation [57], indicating that the human pancreas has at least some degree of regenerative capacity. Unlocking that potential could lead to effective treatment options for diseases such as diabetes and pancreatitis. However, to provide us a better understanding of human pancreatic regeneration, it is imperative to understand the mechanisms underlying organ maintenance, starting with the cell types involved and the signalling pathways responsible.

Long-term organoid cultures of adult pancreatic tissue fragments largely consist of cells expressing ductal markers such as KRT19, SOX9, and MUC1, suggesting that the organoid-forming cells are derived from the pancreatic duct compartment or through transdifferentiation of other pancreatic cells. But what subpopulation of cells exactly forms complex organoids is still unclear. Multiple groups have used cell surface markers or other isolation techniques in an effort to find adult pancreatic exocrine cells capable of clonal expansion in 3D culture. Initially the pan-epithelial cell surface marker EpCAM in combination with the fluorescent chelator TSQ, which binds endocrine granules, was used on adult mouse pancreas to sort EpCAM-positive, non-endocrine epithelial cells that were capable of long-term clonal expansion in 3D culture [36]. By using transgenic mice to lineage trace and sort SOX9 and PTF1a, the authors were able to demonstrate that only SOX9-positive ductal cells were capable of long-term clonal expansion of single cells, whereas PTF1A-positive acinar cells failed to clonally expand over prolonged time.

The cell surface marker CD133 (prominin-1), initially recognized as a surface marker for hematopoietic and neural stem cells [58, 59], has been successfully used to enrich for cells with stem cell or cancer-initiating characteristics from multiple organs [60–64]. In the human fetal and adult pancreas, CD133 has been identified as a cell surface marker labelling ductal cells [65, 66]. Lee et al. [37] used human pancreata to sort CD133-positive single ductal cells that could subsequently be clonally expanded as cystic structures in Matrigel with expansion medium supplemented with Wnt signalling factors. During expansion, these 3D cultures retained some of the characteristics of the primary human pancreatic ducts, such as cell polarity and KRT19 expression. Although in vitro differentiation protocols were applied, expanded ductal cells could not be converted to beta-like cells without adenoviral-mediated overexpression of the beta cell–specific transcription factors MAFA, PDX1, NGN3, and PAX6 followed by a 2-week culture for maturation. Other groups have used CD133 in combination with other cell surface markers to enrich for ductal subpopulations from adult mouse pancreas. For example, CD133(+) cells combined with CD71(low) positivity enriched for an adult mouse pancreas ductal cell subpopulation which could be expanded as cystic structures in 3D culture. When transplanted in diabetic mice, these cells yielded ductal cells, acinar cells, and insulin and glucagon monohormonal cells [67, 68].

The exocrine compartment of the pancreas is for a large part composed of acinar cells. There is limited evidence from rodent studies that suggests that the adult acinar compartment can generate new exocrine and endocrine cells during pancreatic injury [69, 70]. Wollny et al. used cellular size to separate acinar cells from ductal cells in the adult mouse pancreas, and applied a sorting strategy to isolate doublets and triplets of acinar cells from mouse pancreas. They identified a progenitor-like acinar cell subpopulation capable of generating organoids that underwent an acinar-to-ductal metaplasia with a loss of amylase expression and gain of KRT19 expression, indicating that although most acinar cells cannot proliferate or exhibit a limited capacity for expansion in vitro, there are a small subset of acinar cells that retain a long-term but unipotent capacity for expansion [71].

Altogether, these results indicate that enrichment for cell subpopulations with progenitor cell characteristics from the heterogeneous pancreatic exocrine compartment can yield cells with an increased capacity for clonal expansion. However, the exact location of these subpopulations and their role in organ maintenance during homeostasis or injury has not been elucidated.

### Can Pancreatic Organoid Cells Differentiate Towards Insulin-Producing Cells?

Since the organoid culture methods outlined here allow the long-term culture of pancreatic cells, it is now possible to use these as model to further study the mechanisms involved in adult pancreas regeneration. As only 1–2% of the pancreas is composed of endocrine cells and the remaining 98–99% of exocrine cells, insight in the processes that allow endocrine cell specification from exocrine cells could be beneficial for the creation of novel beta cells—this could be used for beta cell replacement therapy for patients with diabetes mellitus, a treatment which is currently hampered due to the shortage of donor tissue. We have demonstrated that the complex organoids derived from human adult islet-depleted tissue fragments harbor cells that have the potential for endocrine differentiation as they can develop into a limited number of hormone positive cells when transplanted in vivo under the kidney capsule of mice [39••]. The required signals that are necessary for this differentiation are not clear, and in vitro endocrine differentiation of human pancreatic organoids is still inefficient.

In order to improve our understanding of the signals required for progenitor cell growth and differentiation, more defined culture medium in organoid models should be used. A serum-free, conditioned medium–free 3D culture model for adult murine pancreatic progenitors was developed by using 7 defined growth factors and small molecules in culture, although extracellular matrix was formed by a mix of methylcellulose and Matrigel. These growth factors combined with ECM were shown to support self-renewal and in vitro differentiation of cells that resemble ductal, acinar, and endocrine cells on the gene expression level, indicating tri-lineage differentiation potential of these multipotent cells [72].

Investigations into the underlying molecular mechanism that enhance endocrine differentiation could provide additional insights into the required factors for endocrine differentiation. Azzarelli et al. [73] used adult mouse pancreatic ductal organoids and demonstrated that the inhibition of post-translation NGN3 protein phosphorylation enhanced stability of the transcription factor, thus promoting increased expression of its downstream target genes, known to drive endocrine differentiation.

The current protocols for the formation of mature and functional beta cells from adult pancreatic organoids are still far from clinical application due to limitations in efficiency, reproducibility, and scalability. Nonetheless, the in vitro platform that organoid culture offers will allow researchers in the field to further decipher the pathways and mechanisms controlling exocrine and endocrine differentiation, which could subsequently aid in the development of future clinical applications such as beta cell replacement therapy. One could envisage that patients undergoing islet autotransplantation after total pancreatectomy due to benign pancreatic disease will receive an additional infusion procedure using their own expanded and differentiated organoids as the isolated islet preparation itself is often insufficient for insulin independence. Naturally this requires more robust expansion and differentiation protocols and also sufficient evidence of genomic stability of these expanded cells before and after transplantation.

### Drug Responses and Toxicity Screening Using Pancreatic Organoids

Pancreatic ductal adenocarcinoma (PDAC) is one of the most lethal forms of cancer with 5-year survival rates less than 8% and is projected to be the second most common cause of cancer-related deaths by 2030 [74, 75]. The high mortality rate of PDAC is largely due to difficulties in early detection, aggressive late-stage metastasis, and large disease heterogeneity resulting in a mixed response to pharmacological treatment [76, 77]. These poor clinical outcomes illustrate the need for new tools to rapidly and accurately identify effective therapies for patients.

Although cell lines, rodent models, and patient-derived xenograft models have generated valuable insights, organoid models have emerged as a superior tool for studying PDAC behavior and assessment of the response to potential therapeutic molecules or screening for drug sensitivities in vitro [78–80]. Romero-Calvo et al. [81] performed detailed comparative analyses by histopathological profiling and thorough genomic characterization by deep sequencing of primary human PDAC tissue, organoids derived from the PDAC tissue and PDAC tissue transplanted in mice. They demonstrated that human PDAC organoids show strong concordance at the structural and genetic level with the primary tumour, and that the genetic composition of PDAC organoids remained constant over multiple passages. Moreover, tumor-specific drug responses could be evaluated in assays, with an in vivo differential drug response to drug treatments of patient-derived xenografts that could be recapitulated in vitro with matched PDAC organoids [81]. These comparative results are in line with findings obtained by other groups that have compared PDAC organoids with the primary tumor and patient-derived xenograft models [38••, 82], and drug sensitivity studies performed by other groups on mouse and human PDAC organoids [38••, 83, 84••]. Novel tools have also been developed to assess drug response of PDAC organoids. Walsh et al. [83] use optimal metabolic imaging (OMI), a novel and non-destructive imaging tool, to quantify drug-induced changes in cellular metabolism in order to evaluate the effect of several anti-cancer treatments on PDAC organoids.

Genomic analysis has identified molecular subtypes of pancreatic cancer and PDAC subtypes as well as their differential response to therapy [85, 86]. PDAC organoids have also been used to study tumor biology and heterogeneity of a single pancreatic tumor. A recent PDAC organoid study used CRISPR gene editing to demonstrate that Wnt independence is acquired over time by genetic and epigenetic mechanisms [87]. This heterogeneity is reflected in differences in the PDAC organoid culture medium composition, with some groups using culture conditions rich in Wnt [38••] and groups using culture conditions without the addition of Wnt [83, 84••]. These differences illustrate the necessity for continuous improvements of PDAC organoid culture methods in order to compare results [87].

Besides the potential to study tumor behavior and drug responses in human PDAC organoids, another advantage is the possibility to generate large amounts of human PDAC organoids from small amounts of tissue. Using endoscopic ultrasound fine needle aspiration (EUS-FNA) to collect tumor tissue, it has been demonstrated that PDAC organoids can be robustly generated with high rates of success with this technique [83, 88]. This enhances the clinical application potential and increases the number of patients that could possible benefit from drug screening and personalized medicine.

Pre-stages of PDAC have also been studied using organoid models. Transplantation of human PDAC organoids into immunodeficient mice resulted in PDAC precursor lesions called pancreatic intraepithelial neoplasms (PanINs) [38••]. This allows us to study the disease progression of these precursor lesions to PDAC and could enable us to identify novel biomarkers and diagnostic opportunities for early stages of invasive PDAC.

In summary, these recent studies demonstrate that large numbers of PDAC organoids can be derived from small tissue samples and that PDAC organoids show great promise for the recapitulation of PDAC tumor characteristics in vitro. A current limitation of the PDAC organoid cultures is the limited recapitulation of tumor microenvironment in vitro, which is provided by stromal cells, blood vessels, nerves, and immune cells in vivo. This tumor microenvironment in PDAC is highly dynamic; promotes tumor progression, metastatic niche formation, and therapeutic resistance; and thus has impact on clinical outcome [89]. Future PDAC organoid studies can address this issue by developing co-culture systems with aforementioned supporting cells [90, 91]. Little information is available on organoid formation from other types of pancreatic tumors. Ultimately investigations into pancreatic cancer organoids will result in better understanding of pancreatic cancer biology and the development of novel, personalized treatment and diagnostic approaches for this disease.

Pancreatic organoids are also a promising tool for screening of toxic effects of drugs and other compounds, such as alcohol, on pancreatic cells. Alcohol is a major cause of acute and chronic pancreatitis in man, but little information is available on the molecular mechanisms that underly this pancreatic disease. Currently, the effects of alcohol have been largely studied on cell lines that exhibit features of acinar cells or on primary acinar cells isolated from rodents [92, 93]. However, these screens are labor intensive and largely limited by quantity of non-expanded primary tissue. Organoids that mimic exocrine functionality can potentially serve as a substitute as they allow for scalability for toxicity screening of large numbers of compounds on organoids with different genetic backgrounds.

### Modelling Monogenetic Diseases with Pancreatic Organoids

Pancreas disease modelling in a 3D culture system can also be performed using induced pluripotent stem cells from subjects with monogenetic diseases. Cystic fibrosis (CF) is an autosomal recessive disease caused by mutations in the cystic fibrosis membrane conductance regulator (CFTR), affecting anion transport and fluid secretion in the pancreas and ultimately leading to chronic pancreatitis and pancreatic insufficiency. Also, diabetes often occurs in patients with CF and pancreas exocrine insufficiency, which is the result of a complex cascade of events that has not been elucidated. Unfortunately, most of the animal models of CF do not fully recapitulate important disease aspects of human CF [94]. Hence, there is a clinical need for better understanding of the effects of CF on the pancreas.

Primary human intestine and rectal tissue from patients with CF have already been cultured as organoids, which could be used for quantitative assays of CFTR function and for the testing of new drugs that modulate CFTR protein function [95, 96]. For the generation of pancreatic organoids with a CFTR gene mutation, iPSC derived from a patient with CF were differentiated to a pancreatic progenitor stage and cultured in Matrigel. In Matrigel, these cells rapidly formed cystic organoids that expressed a wide range of ductal markers, such as KRT19, CFTR, and SOX9 when cultured with FGF2 and nicotinamide. These organoids also showed ductal and acinar cell function, as measured by carbonic anhydrase II (CAII) and enzymes (amylase, trypsin, elastase), and could be used to study the effect of the gene mutation on the structure and behavior of iPSC pancreatic organoids. These models could be applied to further investigate exocrine pancreatic diseases such as cystic fibrosis.

## Conclusion

In conclusion, human pancreatic organoids constitute an important translational bridge between in vitro 2D monolayers composed of a single-cell-type and in vivo animal models. While the expansion and differentiation protocols are still being improved, studies performed with human pancreatic organoids have increased our understanding of pancreas development, homeostasis, and benign and malignant pancreatic diseases. Their potential as a platform for developmental studies, disease modelling, toxicity screening, and personalized treatments for PDAC is already evident, and we envision that they will have a substantial clinical impact in the near future.
